# Review and critical appraisal of studies mapping from quality of life or clinical measures to EQ-5D: an online database and application of the MAPS statement

**DOI:** 10.1186/s12955-018-0857-3

**Published:** 2018-02-12

**Authors:** Helen Dakin, Lucy Abel, Richéal Burns, Yaling Yang

**Affiliations:** 1 Health Economics Research Centre, Nuffield Department of Population Health, University of Oxford, Old Road Campus, Headington, Oxford OX3 7LF UK; 2 Nuffield Department of Primary Care Health Science, University of Oxford, Oxford, UK

**Keywords:** Systematic review, Mapping, EuroQoL, EQ-5D, Utility, Crosswalking, Health-related quality of life

## Abstract

**Background:**

The Health Economics Research Centre (HERC) Database of Mapping Studies was established in 2013, based on a systematic review of studies developing mapping algorithms predicting EQ-5D. The Mapping onto Preference-based measures reporting Standards (MAPS) statement was published in 2015 to improve reporting of mapping studies. We aimed to update the systematic review and assess the extent to which recently-published studies mapping condition-specific quality of life or clinical measures to the EQ-5D follow the guidelines published in the MAPS Reporting Statement.

**Methods:**

A published systematic review was updated using the original inclusion criteria to include studies published by December 2016. We included studies reporting novel algorithms mapping from any clinical measure or patient-reported quality of life measure to either the EQ-5D-3L or EQ-5D-5L. Titles and abstracts of all identified studies and the full text of papers published in 2016 were assessed against the MAPS checklist.

**Results:**

The systematic review identified 144 mapping studies reporting 190 algorithms mapping from 110 different source instruments to EQ-5D. Of the 17 studies published in 2016, nine (53%) had titles that followed the MAPS statement guidance, although only two (12%) had abstracts that fully addressed all MAPS items. When the full text of these papers was assessed against the complete MAPS checklist, only two studies (12%) were found to fulfil or partly fulfil all criteria. Of the 141 papers (across all years) that included abstracts, the items on the MAPS statement checklist that were fulfilled by the largest number of studies comprised having a structured abstract (95%) and describing target instruments (91%) and source instruments (88%).

**Conclusions:**

The number of published mapping studies continues to increase. Our updated database provides a convenient way to identify mapping studies for use in cost-utility analysis. Most recent studies do not fully address all items on the MAPS checklist.

**Electronic supplementary material:**

The online version of this article (10.1186/s12955-018-0857-3) contains supplementary material, which is available to authorized users.

## Background

Recent years have seen increasing numbers of economic evaluations of health technologies. Health technology assessment organisations, including the UK National Institute for Health and Care Excellence (NICE), typically request that economic evaluations should calculate the incremental cost per quality-adjusted life year (QALY) gained [1]. QALYs combine both quantity (i.e. survival) and (health-related) quality of life into a single index. The calculation of QALYs requires health utility data on a 0 to 1 scale where 0 indicates ‘being dead’, 1 indicates ‘full health’ and negative values indicate states ‘worse than death’. NICE also state that the EuroQoL five-dimension (EQ-5D) questionnaire is the ‘preferred measure of health-related quality of life in adults’ [1] to ensure comparability between studies.

For studies where EQ-5D data are not available, mapping or crosswalking techniques are required to predict EQ-5D based on the outcome measures that are available, which may include clinical symptoms, generic or condition-specific non-preference-based quality of life measures or preference-based measures other than EQ-5D [2]. The development and use of mapping algorithms for use in economic evaluations is an actively developing area of methodological and applied research. Although mapping results in information loss and increased uncertainty and is second best compared with direct EQ-5D measurement, it is frequently the only feasible way to conduct cost-utility analysis in cases where direct evidence is unavailable.

Since economic evaluations frequently inform important reimbursement decisions in health care, researchers and health technology assessment organisations need to be able to quickly identify high-quality mapping studies. Several reviews of mapping studies have been published previously [2–6]. An online database, the Oxford Health Economics Research Centre (HERC) Database of Mapping Studies, was established in 2013 to bring together all mapping algorithms predicting EQ-5D [3]. The database has been widely used by researchers around the world; by July 2017 it had been cited in 50 peer-reviewed articles, reflecting the continuing interest in mapping methods and applications. The five-level EQ-5D questionnaire (EQ-5D-5L) has since been used in numerous studies and new utility value sets have become available [7, 8] highlighting the need to update the mapping database to include any studies mapping onto EQ-5D-5L as well as the increasing numbers of studies mapping to EQ-5D-3L.

The 2013 review identified limitations with sample sizes and the range of regression models explored in mapping studies published before that date but did not include formal quality assessments [3]. The Mapping onto Preference-based measures reporting Standards (MAPS) statement [9] was published in 2015 aiming to ‘improve the clarity, transparency and completeness of reporting of mapping studies’. Although the MAPS Statement is a reporting standard rather than a quality assessment tool, it also aims to address these quality issues by increasing the transparency of the methods used in mapping studies. However, to date, no studies have assessed the extent to which the MAPS statement is followed in practice.

We aimed to update this systematic review of mapping studies [3] and assess the impact of the publication of the MAPS statement on the quality of reporting of mapping studies.

## Methods

We updated our previous systematic review [3] to identify studies mapping to EQ-5D. We searched Medline (via Pubmed), EuroQoL Reference Search (www.euroqol.org), the School of Health and Related Research Health Utilities Database (ScHARRHUD, http://www.scharrhud.org), the Centre for Reviews and Dissemination (CRD, www.crd.york.ac.uk/crdweb) database (which includes DARE, NHS EED and HTA) and the Health Economists’ Study Group website (www.hesg.org.uk). The original searches conducted in December 2012 were updated five times between July 2013 and January 2017. We also searched the programmes for the European Association of Health Economics (EuHEA) conference in 2017 and International Health Economics Association (iHEA) conferences in 2013 and 2015. Search terms included “EQ-5D” alongside “mapping”, “mapped”, “crosswalk” or “transfer to utility”. We also reviewed the reference lists of published systematic reviews [2, 4, 6, 10–12]. An additional file gives full details of the searches conducted [see Additional file 1].

We applied the same inclusion/exclusion criteria used previously [3]:

- Conduct statistical mapping (including, but not limited to, regression analyses) to predict EQ-5D utilities and/or responses from any source instrument. We excluded studies using judgement mapping, in which researchers, experts or patients make judgements about how EQ-5D items relate to those on the source instrument. Studies that simply reported the mean EQ-5D utility for different subgroups categorised by the source instrument were excluded unless such data were specifically reported with the intention of being used for mapping.
- Report algorithms or coefficients in sufficient detail that other researchers can use them to predict EQ-5D utilities and/or responses in other studies.
- All versions, tariffs and translations of EQ-5D (including EQ-5D-3L and EQ-5D-5L) were included in the review.
- Papers validating an existing mapping algorithm or developing tools to estimate predictions were identified and linked to the source study in the online database but were not counted as separate studies unless they also estimated new mapping models not reported previously. Similarly, early versions of articles (e.g. conference or discussion papers) meeting inclusion criteria were not counted separately unless they presented additional mapping algorithms; instead such studies are linked to the final version in the online database.
- Valuation studies that did not map to EQ-5D and instead valued health states from the source instrument (e.g. using visual analogue, time trade-off or standard gamble) were excluded.
- Published or made available by 31 December 2016. No other restrictions by publication date or status were imposed providing that the article was available in English.

Details of each study were extracted into the data extraction table used previously [3], using the same methods. The majority of papers identified since our previous paper [3] were extracted by a different reviewer from the one who did the initial screening, providing a second check against the inclusion criteria. Papers that did not clearly meet the inclusion or exclusion criteria were reviewed by all authors and inclusion was decided by consensus. We present results both by paper (or set of related papers) and by mapping algorithm, where an algorithm is defined as a method of mapping from one source measure (or one set of measures) to EQ-5D using a given data set.

### MAPS assessments of reporting quality

The MAPS statement consists of a 23-item checklist of recommendations, intended to improve consistency and transparency in the reporting of mapping algorithms, and hence improve usability and reproducibility [9]. The checklist is organised by section, as found in a standard paper: Title and abstract, Introduction, Methods, Results, Discussion, and additional information, which includes funding and conflicts of interest.

The title and abstracts of all studies meeting the inclusion criteria for the mapping database were assessed for quality using the MAPS statement. We divided the MAPS statement items relating to the title into three sub-items, while those relating to the abstract were broken down into 11 sub-items; an additional file lists the sub-items [see Additional file 2]. Scoring was applied to understand the variation in overall quality by publication year. For each item, abstracts were assigned a score of ‘1’ if the criterion was fulfilled and a score of ‘0’ if it was not fulfilled. Any items that were ‘partially’ completed were assigned a 0.5 scoring. Scores were summed across items to give an overall score out of 11. Four publication time periods were constructed for the analysis of time trends: pre-2011, 2011–2013, 2014–2015 and 2016. The impact of variation in estimated quality scoring across the dichotomous time period variables was examined using ordinary least squares (OLS) regression (assuming the scoring variable was continuous) and ordered logit regression methods (assuming the scoring variable was ordinal). Time trends were modelled using an ordinal variable indicating which of the four time periods each paper was published during (pre-2011 = 1; 2011–2013 = 2; 2014–2015 = 3 and 2016 = 4). The analysis was conducted in Stata version 14.1 (StataCorp, College Station, TX).

In addition to the title and abstract assessment, all included papers published in 2016 were assessed using the full MAPS statement. Each paper and each abstract was independently assessed by one reviewer (ensuring that reviewers did not assess their own work). Reviewers noted whether each criterion was fulfilled (yes, no, partially), the page number or line number, and any comments. For all quality assessments, disagreement surrounding how specific criteria were defined or applied was resolved through discussion.

## Results

### Descriptive statistics

**Fig. 1:**
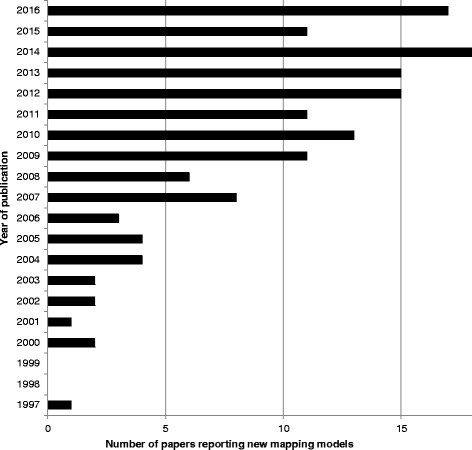
Number of mapping papers by year of publication

In total, 144 studies met the inclusion criteria; these studies included 190 algorithms mapping to EQ-5D [see Additional file 3]. This represents a 62% increase in the number of studies included in the 2013 review [3], which identified 90 studies. The number of studies published each year has remained relatively constant since 2012 (Fig. 1).

The majority of studies, 77%, focused on mapping as the primary objective (111/144) with 9% focusing on methodology (13/144), 8% on economic evaluation (12/144) and 6% on other aspects of quality of life research.

**Fig. 2:**
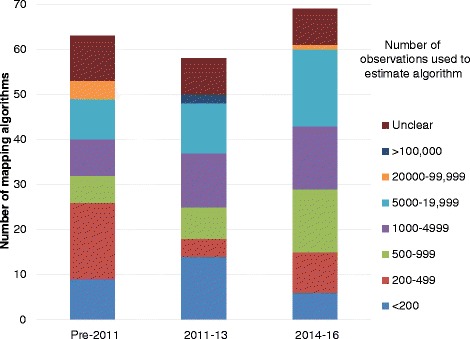
Number of observations used to estimate mapping algorithms by year of publication

Around a third of mapping algorithms (59/190) were estimated on fewer than 500 observations (Table 1). The proportion of mapping algorithms estimated on samples of < 500 observations has halved over time from 41% (26/63) in 1997–2011 to 21% (15/69) in 2014–16 (Fig. 2). However, there was little or no change in the number of algorithms estimated on samples of ≥5000 observations. Furthermore, for 14% (26/190) of mapping algorithms, it was not possible to clearly identify the number of observations used for estimation and the number of algorithms with insufficient information on sample size changed little over time.

**Table 1:** Characteristics of the 190 algorithms mapping to EQ-5D

	Number of mapping algorithms (%)
Number of observations included in estimation sample (including repeated measurements of the same patients)	
< 200	29 (15%)
200–499	30 (16%)
500–999	27 (14%)
1000–4999	34 (18%)
5000–19,999	37 (19%)
20,000–99,999	5 (3%)
> 100,000	2 (1%)
Unclear	26 (14%)
Disease area	
Blood and immune disorders	0 (0%)
Cancer	27 (14%)
Cardiovascular	14 (7%)
Central nervous system	12 (6%)
Digestive system	6 (3%)
Ear, nose and throat	2 (1%)
Endocrine disorders	3 (2%)
Eye conditions	5 (3%)
General population	20 (11%)
Infectious disease	2 (1%)
Mental health and behavioural disorders	9 (5%)
Musculoskeletal	54 (28%)
Pregnancy and childbirth	0 (0%)
Public health	1 (1%)
Respiratory system	9 (5%)
Skin	5 (3%)
Urogenital	2 (1%)
Various	19 (10%)

The review identified studies mapping to EQ-5D from 110 different source instruments or combinations of instruments. Thirty-two algorithms mapped from the health assessment questionnaire (HAQ) to EQ-5D-3L, while 18 mapped from the European Organization for Research and Treatment of Cancer (EORTC) questionnaire; of these 13 algorithms (26%) were published since the 2013 review. Nearly a third of mapping algorithms (53/185) were estimated on patients with musculoskeletal disease (Table 1). By contrast, there were no mapping algorithms focusing on blood and immune disorders or obstetrics.

Four studies (2.8%) mapped to EQ-5D-5L only [13–16], two studies (1.4%) mapped to both EQ-5D-5L and EQ-5D-3L [17, 18], while the remainder mapped to EQ-5D-3L only. Two studies reported algorithms mapping between EQ-5D-3L and EQ-5D-5L [17, 19]. Of the studies mapping to EQ-5D-5L, three [13, 14, 18] used the cross-walk value set [19] and three [15–17] used the EQ-5D-5L value set for England [8]. Furthermore, one study used an experimental version of the EQ-5D-5L questionnaire rather than the final questionnaire approved by the EuroQoL group [13]. No studies mapped either to or from EQ-5D-Y or bolt-on questionnaires.

Simple linear models (e.g. OLS) remain the most commonly used model specification, being explored in the development of 77% (153/190) of mapping algorithms. Tobit was evaluated for 36 algorithms, censored least absolute deviations (CLAD) for 34, generalised linear models for 31 and two-part models for 26 algorithms. Across all years, OLS comprised the only model explored for 34% (64/190) of mapping algorithms, and was compared against other specifications for 46% (87/190) of algorithms; OLS was not evaluated for 21% (39/190) of algorithms. The proportion of studies that only explored OLS models reduced markedly over time from 49% (31/63) in 1997–2011 to 13% (9/69) in 2014–16. Overall, response mapping (in which categorical responses to the five EQ-5D domains are predicted) was explored in the development of 17% (33/190) of the algorithms identified; this proportion rose very slightly from 14% (9/63) in 1997–2011 to 22% (15/69) in 2014–16. Furthermore, mixture models [20] have gained popularity, being used in 12 studies. Since the previous review, several new model specifications have been applied to mapping, including robust MM-estimators [21–23], quantile regression [16, 24], copulas [17] and beta binomial regression [15, 24–26].

### MAPS assessments of reporting quality in titles and abstracts

We assessed the titles of all studies included in the database and compared reporting quality across four time periods (Table 2). Only 55% of studies (79/144) explicitly used the term ‘mapping’ in the title, while 26% (38/144) used a related but non-specific term in the title, such as “predicting”, “comparing”, “deriving” or “estimating”. Of the 27 papers which did not use the term ‘mapping’ or a related-term in the title, only two papers had a primary focus of mapping. Furthermore, only 53% (77/144) of studies stated both the source and target instruments in the title.

**Table 2:** Fulfilment of MAPS assessment of title and abstract^a^

MAPS sub-items	Pre-2011 (*n* = 57)^b^	2011–2013 (*n* = 41)	2014–2015 (*n* = 29)	2016 (*n* = 17)	Overall (%)
Title- used the term ‘mapping’ or synonym	23 (40%)	24 (59%)	19 (66%)	13 (76%)	78 (54%)
Title- indicated ‘source instrument’	34 (60%)	27 (66%)	21 (72%)	11 (65%)	93 (65%)
Title- indicated ‘target instrument’	32 (56%)	28 (68%)	18 (62%)	11 (65%)	89 (62%)
Abstract- Structured abstract	51 (94%)	39 (95%)	29 (100%)	15 (88%)	134 (95%)
Abstract- Objective of mapping stated	41 (76%)	33 (80%)	26 (90%)	14 (82%)	114 (81%)
Abstract- Data sources described	29 (54%)	21 (51%)	19 (66%)	14 (82%)	83 (59%)
Abstract- Source instrument described	46 (85%)	35 (85%)	27 (93%)	16 (94%)	124 (88%)
Abstract- Target instrument described	45 (83%)	37 (90%)	29 (100%)	17 (100%)	128 (91%)
Abstract- Models estimated described	21 (39%)	24 (59%)	20 (69%)	12 (71%)	77 (55%)
Abstract- Other methods described	13 (24%)	13 (32%)	14 (48%)	7 (41%)	47 (33%)
Abstract- Validation strategy described	13 (24%)	11 (27%)	12 (41%)	9 (53%)	45 (32%)
Abstract- Results appropriately reported	39 (72%)	28 (68%)	17 (59%)	11 (65%)	95 (67%)
Abstract- Model performance reported	34 (63%)	23 (56%)	17 (59%)	10 (59%)	84 (60%)
Abstract- Implications of research reported	38 (70%)	33 (80%)	25 (86%)	15 (88%)	111 (79%)
Mean (SD) total abstract score	7.28 (2.93)	7.83 (2.66)	8.83 (1.59)	8.82 (1.77)	7.95 (2.56)

^a^This table highlights the number and proportion of studies in each time period across the MAPS items checklist that fully met each criterion; studies that partially met criteria were included in the total abstract score, but not in the percentage of studies meeting each criterion

^b^Only 54 studies included abstracts that were available for assessment

MAPS was also used to assess abstract quality for the 141 studies with abstracts available (Table 2). The items that were fulfilled by the largest number of studies included a structured abstract (95%), target instruments described (91%) and source instruments described (88%). The items with the lowest compliance included reporting the validation strategy (32%) and giving additional details on analytical methods (33%). Reporting has generally improved over time. For all items apart from having a structured abstract, a higher proportion of papers published in 2016 complied with the MAPS statement compared with the full time period (1997–2016).

**Fig. 3:**
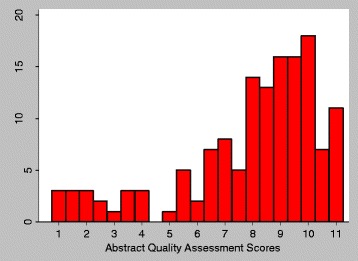
Distribution of quality assessment scoring

**Fig. 4:**
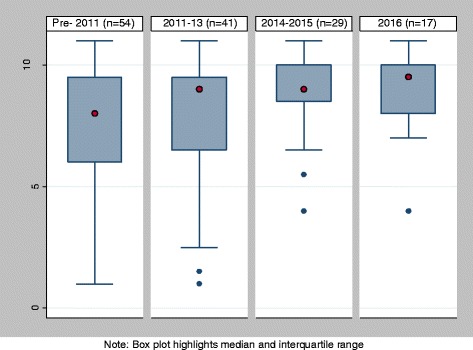
MAPS assessment of title and abstract per year

Scoring for the 11 items on the abstract checklist was applied to all 141 studies with an abstract. The average score overall was 7.95 (SD: 2.55), median was 8.5 (IQR: 7, 10) with a minimum of 1, a maximum of 11 and a mode of 10 (Fig. 3). The mean quality assessment score for abstracts improved significantly over the four time periods assessed when time was measured in an ordinal variable, using both OLS and ordinal logistic regression (*p* < 0.01; Fig. 4). The variation around the quality of reporting has also reduced considerably over time, with the range reducing from 1 to 11 pre-2014 to 4–11 from 2014 onwards.

### MAPS assessments of reporting quality for full papers

Seventeen papers were published in 2016 and assessed using the full MAPS checklist [15, 17, 18, 22, 23, 27–37]; an additional file presents summary statistics of this assessment [see Additional file 2]. This includes two papers that were published online in 2016 and published in print in 2017. Of the 17 papers, one fulfilled all the reporting criteria [28] and one fulfilled all the criteria at least partially [32]. There was wide variation in the quality of reporting between papers. The lowest scoring paper fully reported only five items on the checklist, out of 22. Two papers stated they were following the MAPS reporting guidelines, although neither fulfilled all criteria.

Most criteria were well-reported by a large majority of papers. The criteria that were most commonly well-reported comprised those concerning the study rationale (which all papers reported) and study objective (which was partially reported by three studies and fully reported by all others). Although nine studies included external validation, no paper adequately reported information on the external validation sample they used. Missing data and methods for calculating predicted utilities were also poorly reported. There was mixed performance in how well papers reported their standard errors, validation methods, measures of model performance and exploratory data analysis.

## Discussion

The systematic review identified 144 studies mapping to EQ-5D from 110 different source instruments, including more than 50 new studies that were not included in the previous review [3]. However, one limitation of the review is that due to time and resource constraints, it was not possible to search EMBASE. Furthermore, data extraction and quality assessment were undertaken by single reviewers rather than double-extraction.

This study also assessed how mapping studies have been reported and whether and to what extent the reporting met the standards of the MAPS statement [9]. It provided an overall assessment of how titles and abstracts of mapping studies have been reported over time by applying the title and abstract sections of MAPS checklist to all 141 studies with abstracts. However the full MAPS checklist was only applied to new mapping studies published in 2016 due to time and resource constraints so only a cross-sectional analysis could be conducted.

The analysis of the full text revealed that the standard of reporting was high across most studies, but further improvements are required. The study rationale was universally well-reported among recent papers, and source and target instruments, motivation and context were generally clearly described. However, many studies did not clearly provide all of the information needed to use or reproduce the mapping algorithms, such as the methods for estimating predicted utilities from regression coefficients. The studies mapping to EQ-5D-5L also used a variety of descriptive systems and value sets and did not always clearly report which was used. It is also concerning that 46% of studies (including 24% of papers published in 2016) did not include the word “mapping” in the title, making it difficult for researchers to identify relevant studies; this highlights the importance of systematic reviews bringing together all published studies and suggests that a MESH term for mapping may be helpful.

The quality of titles and abstracts has improved over time, but was already improving in 2014 (before MAPS guidelines were published), so this may reflect an improvement in reporting standards seen for all types of papers. Very few papers reported whether they had chosen to follow the MAPS guidance in their reporting, and those that did so did not score highly on the full text assessment. However, the impact of MAPS may increase in future years: particularly as some 2016 papers will have completed peer-review before the statement was published.

Finally, the MAPS checklist is a reporting standard, rather than an assessment of research quality. Scoring highly on reporting does not necessarily indicate a high-quality study. While good reporting is important to be able to evaluate study quality, it should not be conflated with rigorous analysis. For researchers wishing to evaluate the quality of mapping studies, a number of alternative guidelines have been published. In their overview of mapping for use in NICE health technology assessment, Longworth et al. [2] provided some recommendations on best mapping practice. The recently published ISPOR Good Practices Task Force Report on mapping [38] provides guidance with an international perspective covering all areas of mapping practice, including pre-modelling considerations, modelling and data analysis, reporting of mapping studies, and finally the use of mapping models. While the ISPOR guidelines overlap with MAPS by including 12 items on study reporting, the ISPOR reporting items tend to be more focused on model analysis, do not specify which information should be given in the title, abstract or specific sections and do not provide the level of detail for the reader to easily retrieve the information. In terms of reporting mapping analyses, the MAPS checklist is arguably more comprehensive and easier to use and provides a checklist designed to assess the overall presentation and the necessary components of a mapping paper. As well as considering study and reporting quality, researchers wishing to use a particular mapping algorithm should ensure that the target and source instruments have sufficient conceptual overlap and that the study population reflects their setting.

## Conclusions

The number of mapping studies continues to increase. Reporting of mapping studies has improved over time, although most recent studies failed to fulfil all points on the MAPS checklist. As the MAPS checklist is a recent addition to the mapping literature, it remains to be seen whether it will further increase the reporting quality of future mapping studies over and above the general trend towards improved reporting standards. The update identified the increasing popularity of mixture models and an increasing number of studies mapping to EQ-5D-5L. The database can be used to identify mapping algorithms for use in economic evaluations and other research studies and assess the novelty of mapping studies being planned or undergoing peer review. It is freely available at https://www.herc.ox.ac.uk/downloads/herc-database-of-mapping-studies and will continue to be updated regularly.

## Additional file

Additional file 1: Searches conducted as part of the systematic review. Table summarising the search terms and results for each literature search. (DOCX 43 kb)

Additional file 2: MAPS quality assessment. Comprises one table listing the sub-items used to assess titles and abstracts and one table giving the results of the full text assessment of 2016 papers. (DOCX 46 kb)

Additional file 3: Database of mapping studies version 6 110717. Data extraction table. Spreadsheet copy of the data extraction table, giving details of the studies meeting inclusion criteria. (XLSX 285 kb)

## Abbreviations

CLAD: Censored least absolute deviations
CRD: Centre for Reviews and Dissemination
EORTC: European Organization for Research and Treatment of Cancer
EQ-5D: EuroQol five dimension (3L= three level; 5L= five level; Y= youth)
EuHEA: European Association of Health Economics
HAQ: Health Assessment Questionnaire
HERC: Health Economics Research Centre
iHEA: International Health Economics Association
ISPOR: International Society of Pharmacoeconomics and Outcomes Research
MAPS: Mapping onto Preference-based measures reporting Standards
MeSH: Medical Subject Headings
NICE: National Institute for Health and Care Excellence
OLS: Ordinary least squares
QALY: Quality-adjusted life-year
