# Supplementary material for: Review and critical appraisal of studies mapping from quality of life or clinical measures to EQ-5D: an online database and application of the MAPS statement
Source: Health Qual Life Outcomes. 2018 Feb 12;16:31. doi: 10.1186/s12955-018-0857-3 (PMC5810002; doi:10.1186/s12955-018-0857-3)
Supplement: Supplementary file 1 — Searches conducted as part of the systematic review. Table summarising the search terms and results for each literature search. (DOCX 43 kb) [file 12955_2018_857_MOESM1_ESM.docx]

**Additional file 1: Searches conducted as part of the systematic review**

**Table S1** Searches conducted as part of the systematic review

| **Database** | **Search date** | **Limits** | **Search terms** | **Number of hits** |
| --- | --- | --- | --- | --- |
| ***Version 6: January 2017*** | | | | |
| MEDLINE/PubMed | 17/1/17 | January-December 2016 | (mapping OR map OR mapped OR cross-walk* OR crosswalk* OR "transfer to utility" OR “indirect utility”) AND (eq-5d OR eq5d OR euroqol) | 35 |
| EuroQoL website <http://www.euroqol.org/eq-5d-references/reference-search.html> | 17/1/17 | January 2013-December 2016 | Map OR mapping | 74 |
|  |  |  | crosswalk | 4 |
|  |  |  | “cross-walk” | 0 |
| ScHARRHUD <http://www.scharrhud.org/> | 17/1/17 | Records added since 2016 or published in 2016 | Map* AND EQ-5D | 6 |
|  |  |  | Crosswalk AND EQ-5D | 0 |
|  |  |  | Cross-walk AND EQ-5D | 0 |
|  |  |  | (cross*walk*) AND eq-5d | 0 |
|  |  |  | transfer-to-utility AND EQ-5D | 0 |
|  |  |  | Transfer to utility AND EQ-5D | 0 |
| CRD http://www.crd.york.ac.uk/crdweb/ResultsPage.asp | 17/1/17 | Database entry dates between 20/4/16 and 31/12/2016 | Map* AND EQ-5D | 0 |
|  |  |  | Crosswalk AND EQ-5D | 0 |
|  |  |  | Cross-walk AND EQ-5D | 0 |
|  |  |  | (cross*walk*) AND eq-5d | 0 |
|  |  |  | transfer-to-utility AND EQ-5D | 0 |
|  |  |  | Transfer to utility AND EQ-5D | 0 |
| EuHEA programme for the Hamburg 2017 conference | July 2017 |  | Map | 0 |
| HESG January 2017 Birmingham conference programme | July 2017 |  | Map | 0 |
| HESG June 2016 conference programme | July 2017 |  | Map | 0 |
| ***Version 5: April 2016*** | | | | |
| Medline/Pubmed | 16/04/2016 | Publication dates 2015 onwards | (mapping OR map OR mapped OR cross-walk* OR crosswalk* OR "transfer to utility" OR “indirect utility”) AND (eq-5d OR eq5d OR euroqol) | 52 |
| ScHARRHUD <http://www.scharrhud.org/> | 25/4/16 | None | Map* AND EQ-5D | 23 |
|  |  |  | Crosswalk AND EQ-5D | 1 |
|  |  |  | Cross-walk AND EQ-5D | 0 |
|  |  |  | (cross*walk*) AND eq-5d | 1 |
|  |  |  | transfer-to-utility AND EQ-5D | 0 |
|  |  |  | Transfer to utility AND EQ-5D | 0 |
| CRD http://www.crd.york.ac.uk/crdweb/ResultsPage.asp | 20/4/16 | Database entry dates between 18/7/13 and 20/4/16 | Map* AND EQ-5D | 9 |
|  |  |  | Crosswalk AND EQ-5D | 0 |
|  |  |  | Cross-walk AND EQ-5D | 0 |
|  |  |  | (cross*walk*) AND eq-5d | 0 |
|  |  |  | transfer-to-utility AND EQ-5D | 0 |
|  |  |  | Transfer to utility AND EQ-5D | 0 |
| iHEA: searched program for the Milan 2015 conference abstracts |  |  | map | 1 session |
| HESG January 2016 Manchester conference programme |  |  | map | 0 |
| HESG June 2015 conference programme |  |  | map | 0 |
| HESG January 2015 conference programme |  |  | map | 0 |
| ***Version 4: July 2015*** | | | | |
| MEDLINE/PubMed | 29/7/15 | Any hits with publication dates before 2013 were ignored | (mapping OR map OR mapped OR cross-walk* OR crosswalk* OR "transfer to utility" OR “indirect utility”) AND (eq-5d OR eq5d OR euroqol) | 51 |
| ***Version 3: June 2014*** | | | | |
| MEDLINE/PubMed | 13/6/14 | Any hits with publication dates before 2013 were ignored | (mapping OR map OR mapped OR cross-walk* OR crosswalk* OR "transfer to utility" OR “indirect utility”) AND (eq-5d OR euroqol) | 48 |
| HESG Sheffield January 2014 abstract book |  |  | Map | 1 |
| HESG Glasgow June 2014 abstract book |  |  | Map | 0 |
| ***Version 2: July 2013*** | | | | |
| MEDLINE/PubMed | 18/7/13 | December 2012-July 2013 | (mapping OR map OR mapped OR cross-walk* OR crosswalk* OR "transfer to utility") AND (eq-5d OR euroqol) | 50 |
| EuroQoL <http://www.euroqol.org/eq-5d-references/reference-search.html> | 18/7/13 | Published 2012 or 2013 | Map | 41 |
|  |  |  | crosswalk | 3 |
|  |  |  | Cross-walk | 2 |
| CRD http://www.crd.york.ac.uk/crdweb/ResultsPage.asp | 18/7/13 | Limits to database entry dates between 14/12/12 and 18/7/13 | Map* AND EQ-5D | 6 |
|  |  |  | Crosswalk AND EQ-5D | 0 |
|  |  |  | Cross-walk AND EQ-5D | 0 |
|  |  |  | (cross*walk*) AND (eq-5d) (no date restrictions) | 0 |
|  |  |  | transfer-to-utility AND EQ-5D (no date restrictions) | 1 |
|  |  |  | Transfer to utility AND EQ-5D (no date restrictions) | 1 |
| iHEA abstracts and sessions, Sydney 2013 conference | July 2013 |  | map | 2 sessions |
| HESG conference paper titles, Warwick 2013 |  |  | Map | 1 |
| ***Version 1: December 2012 and April 2013*** | | | | |
| MEDLINE/PubMed | 14/12/12 | None | mapping eq-5d | 64 |
|  |  |  | Mapped eq-5d | 18 |
|  |  |  | Cross-walk* EQ-5D | 1 |
|  |  |  | transfer to utility eq-5d | 1 |
|  |  |  | (mapping OR map OR mapped OR cross-walk* OR crosswalk* OR "transfer to utility") AND (eq-5d OR euroqol) | 88 |
| EuroQoL www.euroqol.org | 8/4/13 | None | Map | 107 |
|  |  |  | crosswalk | 3 |
|  |  |  | Cross-walk | 1 |
| HESG website www.hesg.org.uk | 12/12/12 | None | Mapping | 0 |
|  |  |  | Map | 10 |
|  |  |  | Mapped | 0 |
| CRD www.crd.york.ac.uk/crdweb | 14/12/12 | None | Mapping AND EQ-5D | 15 |
|  |  |  | Map* AND EQ-5D | 25 |
|  |  |  | Crosswalk AND EQ-5D | 0 |
|  |  |  | Cross-walk AND EQ-5D | 0 |
