# Supplementary material for: Review and critical appraisal of studies mapping from quality of life or clinical measures to EQ-5D: an online database and application of the MAPS statement
Source: Health Qual Life Outcomes. 2018 Feb 12;16:31. doi: 10.1186/s12955-018-0857-3 (PMC5810002; doi:10.1186/s12955-018-0857-3)
Supplement: Supplementary file 2 — MAPS quality assessment. Comprises one table listing the sub-items used to assess titles and abstracts and one table giving the results of the full text assessment of 2016 papers. (DOCX 46 kb) [file 12955_2018_857_MOESM2_ESM.docx]

**Additional file 2: MAPS quality assessment**

**Table S2** Methods for quality assessment of titles and abstracts

| **MAPS Title and Abstract Assessment Criteria** | |
| --- | --- |
| **Section** | **Assessment** |
| Title  (3 sub-items) | Identify the report as a mapping study: give the term used (e.g. mapping, crosswalk) |
|  | State source measure(s)? |
|  | State generic target measure(s)? |
| Abstract  (11 sub-items) | Structured Abstract? |
|  | Objectives of the mapping study stated? |
|  | Data sources and their key characteristics described? |
|  | State source measure(s)? |
|  | State generic target measure(s)? |
|  | State the models estimated? |
|  | Give other details of estimation strategy? |
|  | Describe the validation strategy in the abstract? |
|  | Give results in the abstract? |
|  | Describe model performance in the abstract? |
|  | Describe implications of the mapping study in the abstract? |

**Table S3** Results of the quality assessment of the full texts of 2016 papers, showing number of responses recorded out of 16 papers assessed

| **Section/Topic** | **Recommendation** | **Number of responses recorded** | | | |
| --- | --- | --- | --- | --- | --- |
|  |  | **Yes** | **Partly** | **No** | **Not applicable** |
| **Introduction** | | | | | |
| Study rationale | Describe the rationale for the mapping study in the context of the broader evidence base. | 17 | 0 | 0 | 0 |
| Study objective | Specify the research question with reference to the source and target measures used and the disease or population context of the study. | 14 | 3 | 0 | 0 |
| **Methods** | | | | | |
| Estimation sample | Describe how the estimation sample was identified, why it was selected, the methods of recruitment and data collection, and its location(s) or setting(s). | 12 | 5 | 0 | 0 |
| External validation sample | If an external validation sample was used, the rationale for selection, the methods of recruitment and data collection, and its location(s) or setting(s) should be described. | 0 | 3 | 6 | 8 |
| Source and Target measures | Describe the source and target measures and the methods by which they were applied in the mapping study. | 13 | 4 | 0 | 0 |
| Exploratory data analysis | Describe the methods used to assess the degree of conceptual overlap between the source and target measures. | 4 | 6 | 7 | 0 |
| Missing data | State how much data were missing and how missing data was handled in the sample(s) used for the analyses. | 5 | 6 | 6 | 0 |
| Modelling approaches | Describe and justify the statistical model(s) used to develop the mapping algorithm. | 13 | 3 | 1 | 0 |
| Estimation of predicted scores or utilities | Describe how predicted scores or utilities are estimated for each model specification. | 7 | 1 | 9 | 0 |
| Validation methods | Describe and justify the methods used to validate the mapping algorithm. | 10 | 1 | 4 | 2 |
| Measures of model performance | State and justify the measure(s) of model performance that determine the choice of the preferred model(s) and describe how these measures were estimated and applied. | 9 | 5 | 3 | 0 |
| **Results** | | | | | |
| Final sample size(s) | State the size of the estimation sample and any validation sample(s) used in the analyses (including both number of individuals and number of observations). | 14 | 1 | 2 | 0 |
| Descriptive information | Describe the characteristics of individuals in the sample(s) (or refer back to previous publications giving such information). Provide summary scores for source and target measures, and summarise results of analyses used to assess overlap between the source and target measures. | 11 | 5 | 1 | 0 |
| Model selection | State which model(s) is(are) preferred and justify why this(these) model(s) was(were) chosen. | 12 | 4 | 1 | 0 |
| Model coefficients | Provide all model coefficients and standard errors for the selected model(s). Provide clear guidance on how a user can calculate utility scores based on the outputs of the selected model(s). | 10 | 6 | 1 | 0 |
| Uncertainty | Report information that enables users to estimate standard errors around mean utility predictions and individual-level variability. | 10 | 0 | 7 | 0 |
| Model performance and face validity | Present results of model performance, such as measures of prediction accuracy and fit statistics for the selected model(s) in a table or in the text. Provide an assessment of face validity of the selected model(s). | 10 | 6 | 1 | 0 |
| **Discussion** | | | | | |
| Comparisons with previous studies | Report details of previously published studies developing mapping algorithms between the same source and target measures and describe differences between the algorithms, in terms of model performance, predictions and coefficients, if applicable. | 9 | 4 | 2 | 2 |
| Study limitations | Outline the potential limitations of the mapping algorithm. | 15 | 1 | 1 | 0 |
| Scope of applications | Outline the clinical and research settings in which the mapping algorithm could be used. | 10 | 6 | 1 | 0 |
| **Other** | | | | | |
| Additional information | Describe the source(s) of funding and non-monetary support for the study, and the role of the funder(s) in its design, conduct and report. Report any conflicts of interest surrounding the roles of authors and funders. | 11 | 4 | 2 | 0 |
